# On the effects of cycloheximide on cell motility and polarisation in *Dictyostelium discoideum*

**DOI:** 10.1186/1471-2121-7-5

**Published:** 2006-01-24

**Authors:** Margaret Clotworthy, David Traynor

**Affiliations:** 1 MRC Laboratory of Molecular Biology, Hills Road, Cambridge, CB2 2QH, England

## Abstract

**Background:**

Cycloheximide is a protein synthesis inhibitor that acts specifically on the 60S subunit of eukaryotic ribosomes. It has previously been shown that a short incubation of *Dictyostelium discoideum *amoebae in cycloheximide eliminates fluid phase endocytosis.

**Results:**

We found that treatment with cycloheximide also causes the amoebae to retract their pseudopodia, round up and cease movement. Furthermore, fluid phase endocytosis, phagocytosis and capping cease in the presence of 2 mM cycloheximide, although membrane uptake, as measured using FM1-43, is unaffected. In the presence of cycloheximide, aggregation-competent amoebae sensitive to cAMP, although round, can still localise CRAC, ABP120, PI3K and actin polymerisation in response to a micropipette filled with cAMP. The behaviour of wild-type amoebae in the presence of cycloheximide is surprisingly similar to that of amoebae having a temperature-sensitive version of NSF at the restrictive temperature.

**Conclusion:**

Our results may suggest that, upon cycloheximide treatment, either a labile protein required for polarised membrane recycling is lost, or a control mechanism linking protein synthesis to membrane recycling is activated.

## Background

Using *Dictyostelium discoideum *amoebae, Gonzalez and Satre [1] investigated the effect of protein synthesis inhibition on fluid phase uptake. They found that cycloheximide, which acts specifically on the 60S subunit of eukaryotic ribosomes [2], was the most potent inhibitor. Treatment of amoebae with cycloheximide for 30 minutes essentially eliminated fluid phase uptake, as measured using FITC-dextran. This, coupled with their observation that several other inhibitors of protein synthesis have a similar effect, suggested that a labile protein is essential for endocytosis.

We examined *Dictyostelium *amoebae after treatment with cycloheximide and confirmed the observations of Gonzalez and Satre [1]. Furthermore, we noticed that under these conditions the cells withdrew their pseudopodia and rounded up. The loss of NSF (N-ethyl maleimide-Sensitive Factor) also leads to *Dictyostelium *amoebae rounding up and ceasing to move or endocytose fluid phase [3]. NSF is an essential ATPase required for SNARE complex dissociation after membrane fusion events [4-6]. As SNARE proteins are widely required to mediate specific vesicle fusion with the target membrane [7], it is believed that NSF is essential for many vesicular trafficking events, including those at several different stages of the endocytic cycle. The close similarities that were apparent led us to investigate the extent to which the effects of cycloheximide mirrored the mutant NSF phenotype.

Besides examining different aspects of endocytosis, we also studied the ability of the amoebae to polarise various markers. Starving amoebae use cAMP pulses as a chemoattractant during aggregation. Binding of cAMP to its G-protein coupled receptor leads to the translocation of PI3K (Phosphatidyl Inositol-3-OH Kinase) to the membrane at the leading edge of a moving cell. By contrast, PTEN (phosphatidyl inositol 3-phosphatase) is translocated from the cytoplasm to the plasma membrane towards the rear of the cell. The combined uneven distribution of these two enzymes is thought to lead to a local increase in PIP2 and PIP3 levels at the leading edge of the cell, enabling specific PH domain containing proteins, such as CRAC (Cytosolic Regulator of Adenylyl Cyclase) to be recruited there. These steps have been followed in cycloheximide-treated cells using CFP-labelled PI3K [8] and GFP-labelled CRAC [9]. In addition, we also sought to find out whether cycloheximide affects the ability of the cell's cytoskeleton to respond to cAMP signals by following actin polymerisation and its localisation using GFP-tagged actin [10] and the actin-binding domain of ABP120 (Actin Binding Protein 120) [11].

In short, this work explores a possible link between protein synthesis and cell movement.

## Results

### Fluid phase uptake but not membrane uptake is inhibited by cycloheximide

**Figure 1:**
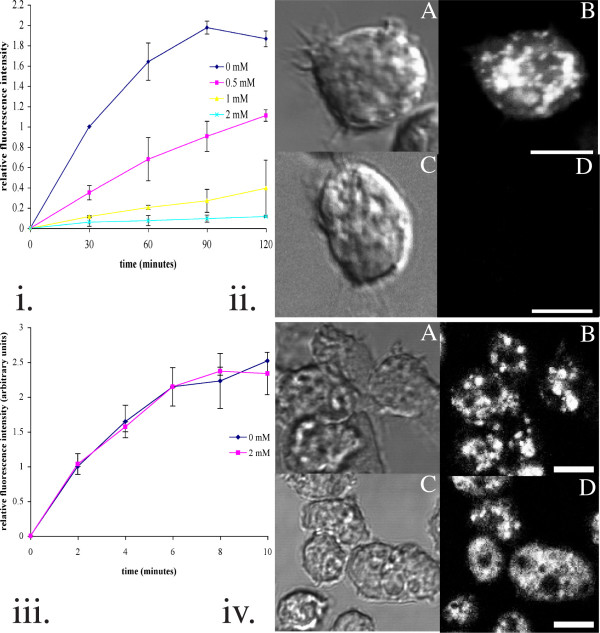
(i) Fluid phase uptake monitored using FITC-dextran. 2 mM cycloheximide inhibits fluid phase uptake by >95%, as shown in this time course. The data obtained for each experiment were normalised with respect to the 30 minute time point for Ax2 in the absence of cycloheximide. (ii) Photomicrographs of amoebae incubated with FITC-dextran. Ax2 were preincubated in the absence (A/ B) or presence (C/ D) of 2 mM cycloheximide for 30 minutes, followed by incubation with FITC-dextran. The amoebae were subsequently fixed in 5% formaldehyde and mounted on a coverslip. A, C: Phase-contrast; B, D: Fluorescence. (iii) Time course showing how membrane uptake, as monitored using FM1-43, is unaffected by cycloheximide. FM1-43 was added at a concentration of 5 μM. The data obtained for each experiment were normalised with respect to the 30 minute time point for Ax2 in the absence of cycloheximide. (iv) Internal membrane bound structures stained with FM1-43. Ax2 were preincubated for 30 minutes without (A/B) or with (C/D) 2 mM cycloheximide prior to incubation with FM1-43 for 60 minutes. The cells were subsequently washed and kept on ice before being placed on a coverslip. A, C: Phase-contrast; B, D: Fluorescence. All images are sections taken using a 60× objective lens unless otherwise stated.

Gonzalez and Satre [1] showed that inhibiting protein synthesis in *Dictyostelium *inhibits fluid phase uptake. We first confirmed this observation with our strain of Ax2: the degree of inhibition was dose dependent (Fig. 1(i) and 1(ii)). The rate of fluid phase uptake over the first 60 minutes ranged from approximately 0.029 to 0.15 μl/1 × 10^7 ^cells/ minute for Ax2 in the absence of cycloheximide. This compares reasonably well with the value of approximately 0.01 μl/1 × 10^6 ^cells/ minute obtained by Vogel et al [12]. In the presence of 2 mM cycloheximide, this was reduced to between 0.38 and 1.7 nl/1 × 10^7 ^cells/ minute.

We next addressed the question of whether bulk membrane uptake is affected by cycloheximide treatment as well. Although some membrane is taken up by macropinocytic vesicles, this is only a small portion of the total surface area taken up [13]. Membrane uptake can be monitored using the dye FM1-43 [14]. This dye is only weakly fluorescent in aqueous solutions, but fluoresces intensely when partitioned into membranes. FM1-43 cannot cross membranes, so when membrane is endocytosed in vesicles, the fluorescence is trapped in the cell and cannot be washed away [15,16]. Hence, FM1-143 provides a convenient measure of total surface membrane uptake. When we looked at the effect of cycloheximide on FM1-43 uptake, we found, unexpectedly, that it had no effect (Fig. 1(iii) and 1(iv)). The initial rate of membrane uptake, when compared with the surface fluorescence obtained for a population of cells as described in the methods section, indicated that one plasma membrane equivalent could be internalised in approximately 8–10 minutes.

### Capping requires protein synthesis

**Figure 2:**
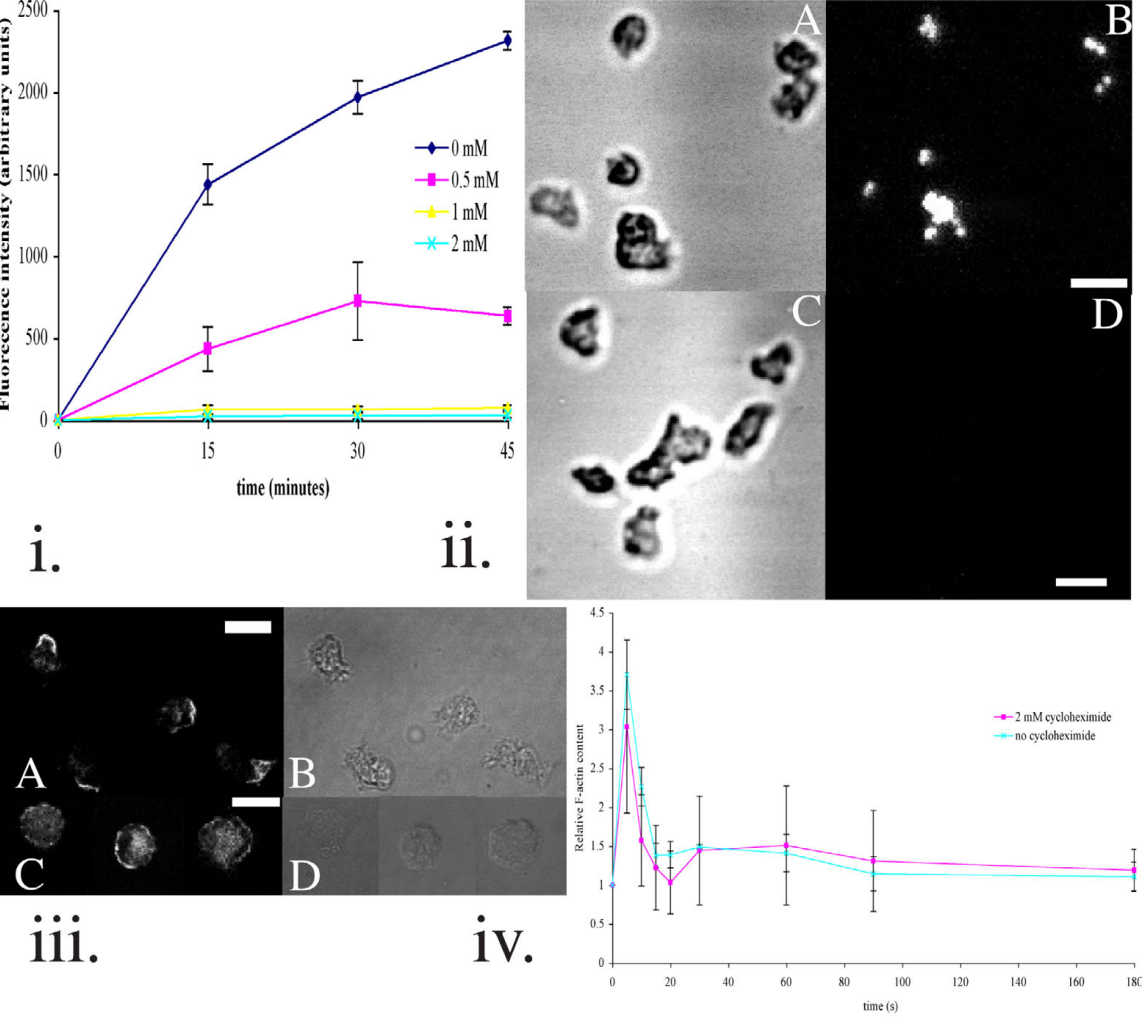
(i) Time course following the phagocytosis of 1 μm beads. Phagocytosis is abolished by 1 mM cycloheximide. (ii) Amoebae preincubated with 2 mM cycloheximide (C/D) do not phagocytose 1 μm beads. A fluorescence reading of approximately 325 units corresponds to an average of 1 bead taken up per cell, a reading of 650 implies that 2 beads have been taken up per cell and so on. Ax2 were preincubated for 30 minutes without (A/B) or with (C/D) 2 mM cycloheximide prior to incubation with fluorescent beads for 30 minutes. The cells were then washed and kept on ice until being allowed to settle on a microscope slide and viewed as sections under 60× magnification on a coverslip. A/C: Phase-contrast image; B/D: Fluorescence image. (iii) 2 mM cycloheximide abolishes capping of Con A receptors cross-linked with fluorescent Con A. Vegetative amoebae settled on glass coverslips were preincubated in the absence (A/B) or presence (C/D) of 2 mM cycloheximide for 30 minutes before being incubated with fluorescent Con A for 1 minute, rinsed and then left in KK2 Mg/ 1 mM Ca^2+ ^for 3–5 minutes prior to fixing in 5% formaldehyde. A, C: fluorescent; B, D: phase-contrast image. All images are sections taken using a 60× objective lens unless otherwise stated. (iv) Actin polymerisation followed using TRITC-phalloidin to stain the fixed actin cytoskeleton in lysed amoebae. The dynamics and relative magnitude of actin incorporation into the triton-insoluble cytoskeleton following stimulation with 1 μM cAMP were found to be similar for control and cycloheximide-treated amoebae.

Capping is the process by which cross-linked antigens or receptors on the cell surface are swept to the rear in migrating cells [17,18]. We used fluorescent tetrameric Con A (Concanavalin A) to cross-link Con A receptors and observed capping of the fluorescence towards the rear of approximately 90% of migrating cells in the absence of cycloheximide (n = 200 amoebae). 99% of cycloheximide-treated cells failed to cap (n = 140 amoebae). Fig. 2(iii) shows a representative sample of treated and untreated amoebae.

### Phagocytosis is sensitive to cycloheximide

Phagocytosis has long been known to involve actin cytoskeleton remodelling [19], and is also thought to require local membrane exocytosis [20,21]. Phagocytosis was monitored by measuring the uptake of 1 μm diameter carboxylate-modified Fluorescent Microspheres in the presence or absence of cycloheximide. The amoebae were preincubated for 30 minutes in various concentrations of cycloheximide prior to addition of the fluorescent beads, and were subsequently shaken for the time intervals shown, before being washed and the fluorescence measured (Fig. 2,(i)). Amoebae in buffer alone were able to phagocytose several beads each over 30 minutes, whilst the cycloheximide-treated amoebae generally contained none (Fig. 2,(ii)).

[see Additional file 3] Fluorescent ABP120-transformed amoebae without cycloheximide

[see Additional file 4] Fluorescent actin-transformed amoebae with cycloheximide

[see Additional file 5] Fluorescent actin-transformed amoebae without cycloheximide

### Cell motility is blocked by cycloheximide treatment

**Figure 3:**
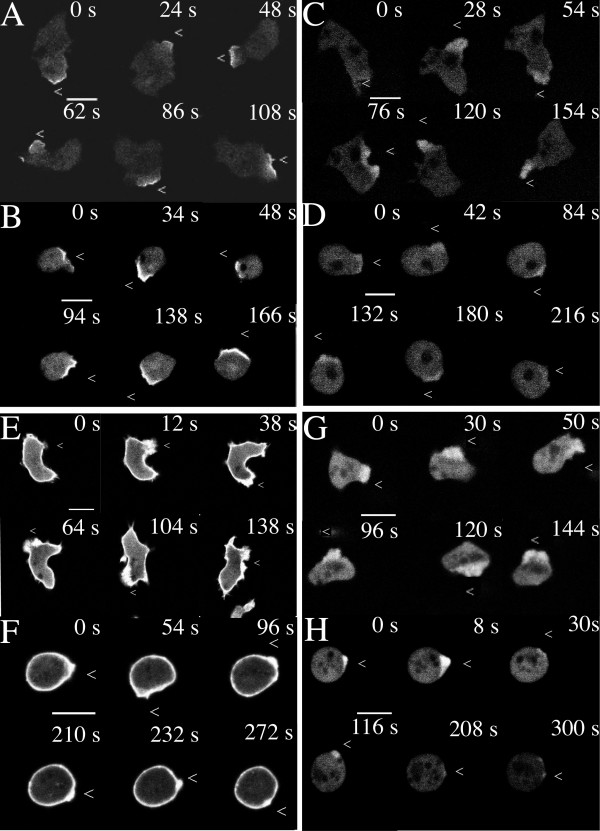
CRAC-GFP (A/B), PI3K2-CFP (C/D), ABP120-GFP (E/F) and Actin-GFP (G/H) can translocate in response to a micropipette filled with 1 μM cAMP in the presence of cycloheximide. Polarisation of these markers was followed in the absence (A, C, E, G) or presence (B, D, F, H) of 2 mM cycloheximide. The position of the cAMP-filled micropipette is indicated by the white "<" symbol. The micropipette always comes in from the right-hand side. Fluorescent images were taken as sections using a 60× objective lens. Scale bar represents 10 μm. For movies, see Additional files.

We observed that vegetative *Dictyostelium *amoebae lost their ability to translocate upon cycloheximide treatment and wanted to investigate whether aggregation competent cells were still able to chemotax towards cAMP. We found that the cells were unable to move but could form small pseudopods in the direction of chemoattractant (Fig. 3). Also, NIH Image was used in conjunction with standard techniques to quantify the differences in speed and persistence between cycloheximide-treated and untreated cells chemotaxing towards a micropipette filled with cAMP. In the absence of cycloheximide, amoebae chemotaxed towards the source of cAMP with an average speed of 12.8 ± 4.6 μm/ minute (n = 30 cells). This compares well with the value of 12.29 ± 2.14 recorded for wild-type cells moving up a gradient [22]. The persistence value for the chemotaxis of these amoebae was 0.74 ± 0.28, over 20 successive images recorded at 20 s intervals (data not shown). A value of 1 represents chemotaxis in a straight line towards the source of cAMP. In the presence of cycloheximide, speed was reduced to 1.2 ± 0.54 μm/ minute (n = 38 cells). For the 3 cells for whom persistence values could be calculated for part of the frame sequence, it was found to be reduced to 0.39 ± 0.36.

We speculated that this loss of speed and persistence could be due to a defect in the cells' ability to translocate polarising signalling molecules such as PI3K and CRAC to the leading edge. The ability of amoebae these proteins was therefore investigated using CFP- and GFP-tagged proteins respectively. Conditioned amoebae were stimulated with a micropipette containing cAMP and we found that the amoebae were able to localise each of the markers towards the chemoattractant, even though the cells were round (Fig. 3 and Additional files). We then considered whether aberrant actin remodelling might be partly responsible for the phenotypes observed.

[see Additional file 6] Fluorescent CRAC-transformed amoebae with cycloheximide

[see Additional file 7] Fluorescent CRAC-transformed amoebae without cycloheximide

[see Additional file 8] Fluorescent PI3K-transformed amoebae with cycloheximide

[see Additional file 9] Fluorescent PI3K-transformed amoebae without cycloheximide

### The rate of Actin polymerisation does not depend on protein synthesis

The dynamics and magnitudes of actin polymerisation were followed using GFP-tagged ABP120 and actin (Fig. 3(E–H) and additional files). We observed that actin did indeed polymerise at the edge of the cell adjacent to, and in response to, the cAMP-filled micropipette, irrespective of whether the cells had been preincubated in the presence of cycloheximide or not. The course of actin polymerisation after cAMP addition was also followed fluorometrically using TRITC-phalloidin to label the triton-insoluble actin cytoskeleton. The kinetics of actin assembly and the relative amounts polymerised were similar whether cycloheximide was present or not (Fig. 2(iv) ).

[see Additional file 2] Fluorescent ABP120-transformed amoebae with cycloheximide

## Discussion

Our starting point was that amoebae treated with cycloheximide ceased fluid phase endocytosis [1], a property also shared with macrophages [23]. In addition, we observed that the amoebae rounded-up and ceased movement, a behaviour surprisingly like that of the NSF temperature-sensitive mutant at the restrictive temperature [3]; this led us to explore further the effects of cycloheximide.

We found that, like fluid phase endocytosis, phagocytosis of latex beads is eliminated by preincubation of amoebae with cycloheximide, a phenomenon which also occurs in monocytes [23,24]. By contrast, surface uptake measured with FM1-43 remains surprisingly unaffected: in fact, the rate of membrane internalised agrees well with the findings of Aguado-Velasco and Bretscher [13]. This indicates that this internalised membrane is endocytosed along with very little fluid and therefore, the vesicles (if that is how the membrane is internalised) must either be flattened or have a very small radius. A similar conclusion comes from studies of FM1-43 uptake in the wild-type amoeba, NC4 [13]. Thus the 5% fluid phase uptake remaining on cycloheximide treatment may correspond to the small amount of fluid inevitably taken up in conjunction with this membrane. Comparison with the ts mutant NSFA2 is also interesting. At the restrictive temperature, NSFA2 is unable to take up fluid phase or phagocytose: in this sense, it is like Ax2 treated with cycloheximide. However, FM1-43 uptake by NSFA2 at the restrictive temperature is reduced (by about 75%), but is not eliminated [3]: however long the mutant is held at the restrictive temperature, there is always a small burst of dye uptake when it is added and this ceases within a few minutes (Thompson and Bretscher, unpublished observations [see Additional file 10]). This uptake may reflect a short circuit endocytic cycle independent of NSF that continues at the restrictive temperature; however, in this case there is no opportunity for the dye to back-fill into other compartments in the cell because these processes presumably require NSF. This contrasts with the effects of cycloheximide, which does not alter membrane uptake appreciably. If the above conjectures are correct, both the short circuit endocytic cycle and back-filling would continue as in the wild type. This is what we observe.

Because amoebae treated with cycloheximide round up and stop moving – much like the NSF ts mutants – we further examined those properties of a cell required for locomotion: an effective motor and a cell polarity. It is a property of all motile cells that they cap cross-linked surface antigens: amoebae are no exception. They cap Con A receptors with great efficiency: by contrast, cycloheximide-treated amoebae do not. By this test, cycloheximide converts these cells from a motile to a non-motile state, suggesting that either the motor or the intrinsic polarity is lost.

To gain further insight into what the effects of cycloheximide are, we tested actin polymerisation in response to cAMP (the "cringe" reaction). Both untreated and cycloheximide-treated amoebae showed similar filament formation in terms of their time courses, and the relative amounts of actin polymerised. Furthermore, the actin cytoskeleton can rapidly reorganise itself in response to a changing source of cAMP, despite the fact that the cells are rounded. Both lines of evidence indicate that the amoebae continue to possess an active cytoskeleton, although (as in the similar case with the NSF ts mutant (3)) some other undetected cytoskeletal defect may exist.

As actin polymerisation occurred in the direction of the cAMP source in cycloheximide-treated cells, it seemed likely that their signal transduction system would function normally in response to an external cAMP signal. That this is so is supported by the observations that PI3K and CRAC both localise towards the cAMP source. In these senses, the cycloheximide-treated cells and the NSF ts mutant share similar characteristics (David Traynor, unpublished observations).

Before turning to how cycloheximide causes these effects, it is relevant to ask how a cycloheximide resistant mutant, HH31, behaves. The rate of fluid phase uptake, as measured using FITC-dextran, was unaltered in the resistant mutant [see Additional file 1]. Additionally, when observed under the microscope, the amoebae remain motile and do not round up (data not shown). The conclusion that the target of our experiments is the ribosome and protein biosynthesis is supported by the other ribosomal inhibitors tested by Gonzalez and Satre [1]: they all behave like cycloheximide.

Therefore, our observations indicate that continued protein synthesis is required for maintaining amoebae in a locomotory state: that may mean that a metabolically unstable protein is required. The similarities in the behaviours of the NSF mutant at the restrictive temperature and cycloheximide-treated cells, with the limited exception of membrane uptake (as discussed above), are striking: cycloheximide appears to phenocopy the NSF mutant.

There seem to be two general categories of interpretation of these observations. It could be that we are seeing a general cellular response to poor growth situations – where protein synthesis or membrane transport fails. This possibility has a precedent: a mechanism appears to exist in yeast that ties cell processes to a continuation of protein synthesis [25]. Alternatively, it could be that an unstable protein is required to maintain the cells in a migratory state, and so is membrane transport. In this latter case, the two lines of evidence might suggest – if they are connected, which they need not be – that an unstable protein(s) is needed to maintain cell motility and that this protein is involved in, or needs to be transported in the cell by, the membrane system.

In the course of this work we noticed that, for clearer results, the length of preincubation with cycloheximide could be varied, depending on the particular assay. For example, fluid phase uptake is blocked 30 minutes after cycloheximide addition, yet 60 minutes is required to block migration. This is not surprising: each of these is a complex process and the dependence on a single factor (if that is what it is) need not be the same for each. A similar phenomenon is seen with the NSF mutant: fluid phase uptake is effectively abolished by 5 minutes at the restrictive temperature, yet cell migration continues quite happily until about 15–20 minutes after the temperature is raised (M.S. Bretscher, personal communication [see Additional file 11]). Indeed, in so far as this comparison has been explored, the assays in which Ax2 is more sensitive to cycloheximide seem to be those in which the NSF mutant is most sensitive.

## Conclusion

Our results show that movement, fluid phase uptake, phagocytosis and capping require continued protein synthesis, whilst localised actin polymerisation, membrane uptake and PI3K and CRAC translocation to the leading edge do not. Our results may suggest that, upon cycloheximide treatment, either a labile protein required for motility and perhaps involving polarised membrane recycling is lost, or a control mechanism linking protein synthesis to membrane recycling is activated.

## Methods

### Strains and culture

Amoebae were shaken at 22°C in axenic medium (supplemented with 0.1 mg/L vitamin B12, 0.02 mg/L biotin and 0.2 mg/L riboflavine and 10 μg/ ml tetracycline) to a density of 1–5 × 10^6 ^cells/ ml. Wild-type Ax2 transfected with constructs to express CRAC-GFP [9], GFP fused to the actin-binding domain of ABP120 [11], PI3K2-CFP [8] or Actin-GFP [10] were cultured in axenic medium supplemented with 5–20 μg/ ml G418. All experiments were performed using vegetative cells cultured in axenic medium unless otherwise stated. Cells were grown axenically prior to use in experiments as it has been documented that amoebae grown axenically endocytose fluid approximately 100 times as fast as amoebae grown on bacteria [26].

### Development

Aggregation-competent amoebae were generated as described by [27] in HEPES-Ca^2+^, pH7. The amoebae were then treated with 5 mM caffeine for 10 minutes. Caffeine is an adenyl cyclase inhibitor and so was used to bring the cells to a basal state of cAMP signalling, making the cells more responsive to the cAMP released from the needle [9]. The amoebae were subsequently washed in fresh buffer without caffeine and resuspended in clean buffer, again without caffeine.

### FITC-Dextran uptake

This assay was performed as described [13], but using MES-Na^+ ^buffer (40 mM MES adjusted to pH 6.5 using NaOH) and incubating vegetative cells at 1 × 10^7 ^cells/ ml with or without cycloheximide for 30 minutes prior to the addition of 2 mM FITC-dextran. 0.4 ml cell suspension were removed at each time point and washed twice in a volume of 1 ml ice cold buffer containing 1% BSA. The resultant pellet was resuspended in 100 μl buffer, transferred to a clean tube and washed once more in 1 ml ice-cold buffer. The zero time point was obtained by chilling a sample of cell suspension on ice prior to the addition of the appropriate volume of FITC-dextran and immediately washing as for the other samples. Samples were lysed in 0.2% Triton X-100 in 100 mM Tris-HCl pH 8.6 and read at excitation wavelength 490 nm, emission wavelength 520 nm.

### FM1-43 uptake

This assay was performed as described by [13] except that the cell density employed was 1 × 10^7 ^cells/ ml in MES-Na^+ ^buffer and the vegetative cells were incubated for 30 minutes with or without cycloheximide prior to the addition of FM1-43 at a final concentration of 5 μM. 0.4 ml samples were removed and washed as described for FITC-dextran uptake. The FM1-43 fluorescence was then determined. The zero time point was obtained by chilling a sample of cell suspension on ice before the addition of the appropriate volume of FM1-43 and immediately washing as for the other samples. In order to determine the fluorescence represented by the internalisation of one plasma membrane equivalent for a population of cells, 5 μM FM1-43 was added to 0.4 ml cell suspension, on ice. This sample was centrifuged, the supernatant was carefully aspirated and the pellet lysed in 1% Triton-X100 and read at excitation wavelength 470 nm, emission wavelength 570 nm as for the other samples. Where photographs of amoebae were to be taken, samples were washed as usual and allowed to settle on coverslips for approximately 10 minutes prior to live imaging. The cells could not be fixed using formaldehyde as this interfered with the quality of the images of the fluorescent structures within the cells.

### Phagocytosis

This assay was performed along the lines described by [28]. Vegetative Ax2 amoebae were shaken at a density of 4 × 10^6 ^cells/ ml in axenic medium for 30 minutes in the presence or absence of cycloheximide prior to the addition of 1 μl of 1 μm FluoSpheres^® ^Fluorescent Microspheres (Molecular Probes) per ml cell suspension, corresponding to a concentration of 2.7 × 10^7 ^beads/ ml. 0.4 ml samples were removed and washed as described for FITC-dextran uptake, and the pellet was resuspended in 1 ml of lysis buffer (as for FITC-dextran uptake) and shaken on a Vibromixer for approximately 15 minutes to disintegrate the cells thoroughly. This avoided the need for centrifugation to remove any remaining debris, as prolonged centrifugation would be likely to also remove the beads from the suspension. The zero time point was obtained by chilling a sample of cell suspension on ice prior to the addition of the appropriate volume of beads and immediately transferring 0.4 ml of the suspension to 1 ml ice-cold KK_2_/ 1% FCS and washing. Samples were read as for FITC-dextran uptake.

### Capping

This assay was performed essentially as described [17]. Vegetative Ax2 were shaken at a density of 1 × 10^7 ^cells/ ml in axenic medium for 30 minutes in the presence or absence of cycloheximide prior to the addition of 15 μg/ ml TRITC-labelled Concanavalin A (Vector Laboratories) for 2 minutes. The amoebae were then removed into microfuge tubes and washed twice in axenic medium. They were then allowed to settle on coverslips where they were incubated for 10 minutes in medium with or without cycloheximide. Finally, formaldehyde was added at 5% as a fixative. DIC and fluorescent images were collected through a 60× (1.4 NA) oil immersion lens on a Nikon Eclipse TE300 microscope fitted with the BioRad Radiance confocal system.

### Polarised localisation of CRAC, PI3K, ABP120, PhdA and Actin polymerisation

Approximately 1 × 10^5 ^aggregation-competent amoebae containing the relevant fluorescently labelled protein were deposited onto the surface (4.2 cm^2^) of a Lab-Tek^® ^(Nalgene) chambered coverglass in 2 ml of MES-Na^+ ^and allowed to settle for approximately 10 minutes. The amoebae were challenged with a micropipette (Eppendorf Femtotips^® ^II) containing 1 μM cAMP (K^+ ^salt, Sigma) using an electronic micromanipulator (Eppendorf 5171) to induce the localised accumulation of the GFP-fusion proteins. DIC and fluorescent images were collected through a 60× (1.4 NA) oil immersion lens on a Nikon Eclipse TE300 microscope fitted with the BioRad Radiance confocal system. Images were taken every 2–5 seconds. Cycloheximide was subsequently added to a final concentration of 2 mM. After 60 minutes, the amoebae were challenged again with the micropipette containing 1 μM cAMP. The amoebae were pre-incubated for 60 minutes for both this and the actin polymerisation assay as it was observed that aggregation-competent cells could take up to 1 hour to round up. Due to the spherical nature of the cells when in cycloheximide, the background fluorescence may appear to be greater than for flat cells as the focus is on a greater depth of cytoplasm.

### Actin polymerisation

Aggregation-competent amoebae were incubated with or without 2 mM cycloheximide for 60 minutes. At the time intervals shown, the F-actin associated with the triton-insoluble cytoskeleton was determined spectroscopically using TRITC-labelled phalloidin [29-31].

## List of abbreviations used

ABP120: Actin Bundling Protein 120

cAMP: cyclic 3',5'-Adenosine MonoPhosphate

CFP : Cyan Fluorescent Protein

Con A : concanavalin A

CRAC : Cytosolic regulator of adenyl cyclase

FCS : bovine Foetal Calf Serum

FITC-dextran : Fluorescein Iso Thio Cyanate – dextran

FM1-43 (SynaptoGreen™) : Frie Mao 1–43 (N-(3-Triethylammoniumpropyl)-4-(p- dibutylaminostyryl) pyridinium dibromide)

GFP : Green Fluorescent Protein

MES : 2-Morpholino Ethane Sulphonic acid

NEM : N-Ethyl Maleimide

NSF : NEM-Sensitive Factor

PH : Pleckstrin Homology

PhdA : Pleckstrin homology domain protein A

PIP2 : PhosphatidylInositol-3,4-diPhosphate

PIP3 : PhosphatidylInositol-3,4,5-triPhosphate

PI3K : PhosphatidylInositol-3-OH Kinase

SNARE: soluble NSF attachment protein receptor

TRITC-Phalloidin : Tetra-methyl Rhodamine IsoThio- Cyanate-Phalloidin

ts : temperature-sensitive

## Authors' contributions

MC performed the assays described and drafted the paper. DT assisted with experimental design.

## Supplementary Material

Additional File 1 Comparison of the effects of cycloheximide on fluid phase uptake, as measured using FITC-dextran, in the wild-type strain Ax2 and a cycloheximide resistant mutant, HH31. Fluid phase uptake is inhibited by 2 mM cycloheximide in Ax2 but not in the cycloheximide-resistant mutant, HH31. Amoebae were incubated in various concentrations of cycloheximide for 30 minutes, followed by the addition of 2 mM FITC-dextran. The data for each experiment were normalised with respect to the 30 minute time point for Ax2 in the absence of cycloheximide. Movies: All movies were taken as sections every 2 seconds, using a 60× (1.4 NA) oil immersion lens on a Nikon Eclipse TE300 microscope fitted with the BioRad Radiance confocal system. The movies are played at 6 frames per second. The movies were generated by merging the DIC (grey) and fluorescence images. The fluorescence intensity is graded from red (low intensity) to white (high intensity) for 3 of the movies, ABP120 with and without cycloheximide, and actin without cycloheximide. For the other movies, the fluorescence is in green and the relative intensity is not indicated. Click here for file

Additional File 2 ABP120 with cycloheximide. Aggregation-competent amoebae were allowed to settle on a chambered coverglass for 10 minutes before being incubated with 2 mM cycloheximide for 1 hour and then stimulated with a micropipette filled with 1 μM cAMP in the presence of cycloheximide. Click here for file

Additional File 3 ABP120 without cycloheximide. Aggregation-competent amoebae were allowed to settle on a chambered coverglass for 10 minutes before being stimulated with a micropipette filled with 1 μM cAMP in the presence of cycloheximide. Click here for file

Additional File 4 Actin with cycloheximide. Aggregation-competent amoebae were allowed to settle on a chambered coverglass for 10 minutes before being incubated with 2 mM cycloheximide for 1 hour and then stimulated with a micropipette filled with 1 μM cAMP in the presence of cycloheximide. Click here for file

Additional File 5 Actin without cycloheximide. Aggregation-competent amoebae were allowed to settle on a chambered coverglass for 10 minutes before being stimulated with a micropipette filled with 1 μM cAMP in the presence of cycloheximide. Click here for file

Additional File 6 CRAC with cycloheximide. Aggregation-competent amoebae were allowed to settle on a chambered coverglass for 10 minutes before being incubated with 2 mM cycloheximide for 1 hour and then stimulated with a micropipette filled with 1 μM cAMP in the presence of cycloheximide. Click here for file

Additional File 7 CRAC without cycloheximide. Aggregation-competent amoebae were allowed to settle on a chambered coverglass for 10 minutes before being stimulated with a micropipette filled with 1 μM cAMP in the presence of cycloheximide. Click here for file

Additional File 8 PI3K with cycloheximide. Aggregation-competent amoebae were allowed to settle on a chambered coverglass for 10 minutes before being incubated with 2 mM cycloheximide for 1 hour and then stimulated with a micropipette filled with 1 μM cAMP in the presence of cycloheximide. Click here for file

Additional File 9 PI3K without cycloheximide. Aggregation-competent amoebae were allowed to settle on a chambered coverglass for 10 minutes before being stimulated with a micropipette filled with 1 μM cAMP in the presence of cycloheximide. Click here for file
