# Supplementary figures and images for: On the effects of cycloheximide on cell motility and polarisation in Dictyostelium discoideum
Source: BMC Cell Biol. 2006 Jan 24;7:5. doi: 10.1186/1471-2121-7-5 (PMC1368985; doi:10.1186/1471-2121-7-5)

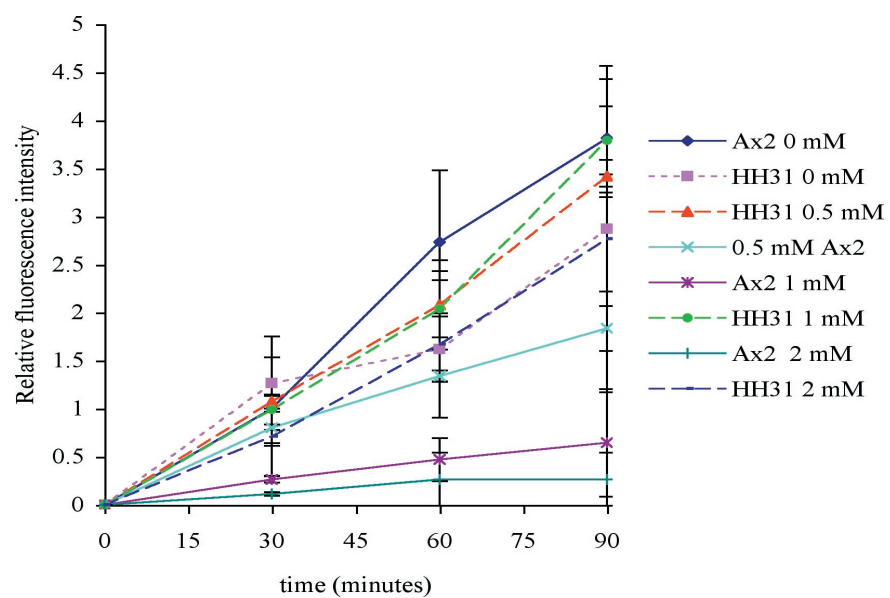

Supplement: Additional File 1 — Comparison of the effects of cycloheximide on fluid phase uptake, as measured using FITC-dextran, in the wild-type strain Ax2 and a cycloheximide resistant mutant, HH31. Fluid phase uptake is inhibited by 2 mM cycloheximide in Ax2 but not in the cycloheximide-resistant mutant, HH31. Amoebae were incubated in various concentrations of cycloheximide for 30 minutes, followed by the addition of 2 mM FITC-dextran. The data for each experiment were normalised with respect to the 30 minute time point for Ax2 in the absence of cycloheximide. Movies: All movies were taken as sections every 2 seconds, using a 60× (1.4 NA) oil immersion lens on a Nikon Eclipse TE300 microscope fitted with the BioRad Radiance confocal system. The movies are played at 6 frames per second. The movies were generated by merging the DIC (grey) and fluorescence images. The fluorescence intensity is graded from red (low intensity) to white (high intensity) for 3 of the movies, ABP120 with and without cycloheximide, and actin without cycloheximide. For the other movies, the fluorescence is in green and the relative intensity is not indicated. [file 1471-2121-7-5-S1.pdf]
